# Supplementary figures and images for: A permutation test and spatial cross-validation approach to assess models of interspecific competition between trees
Source: PLoS One. 2020 Mar 11;15(3):e0229930. doi: 10.1371/journal.pone.0229930 (PMC7065802; doi:10.1371/journal.pone.0229930)

Focal

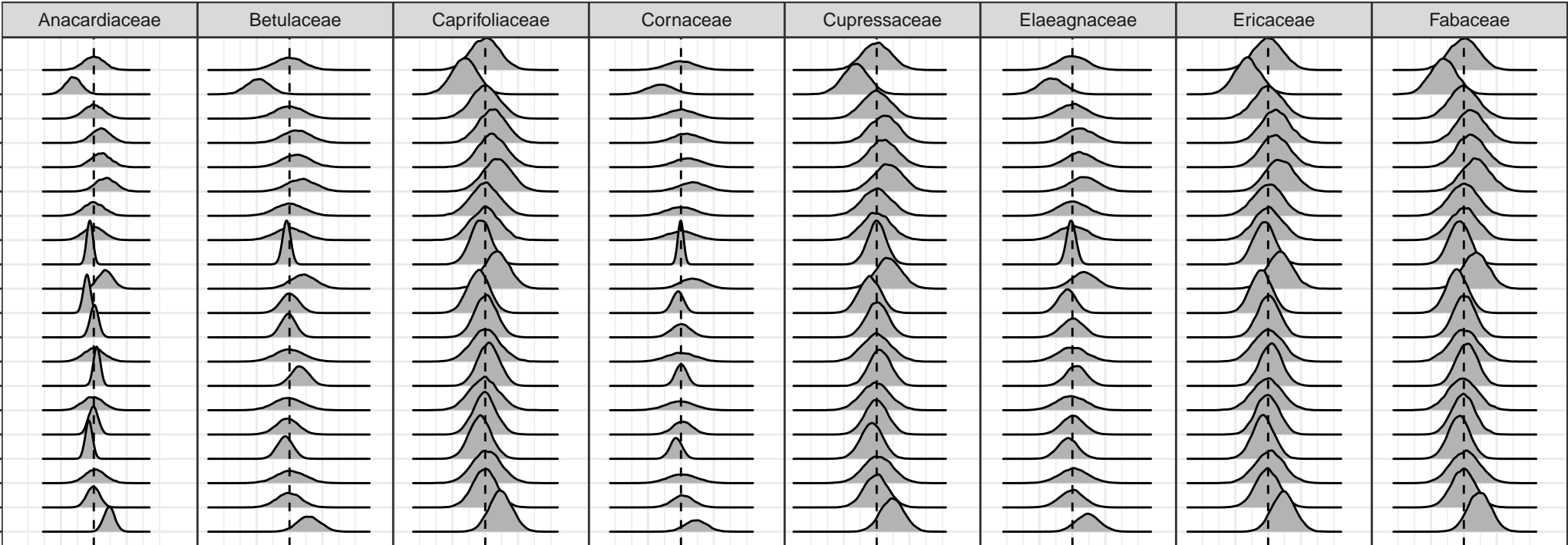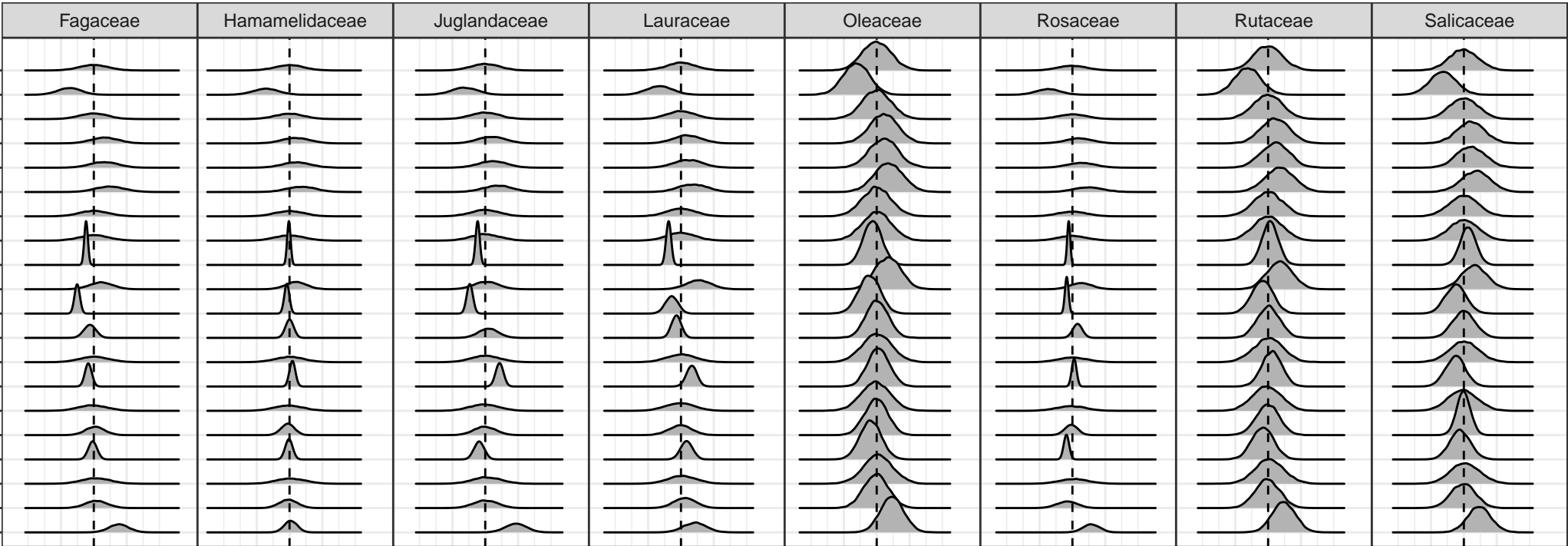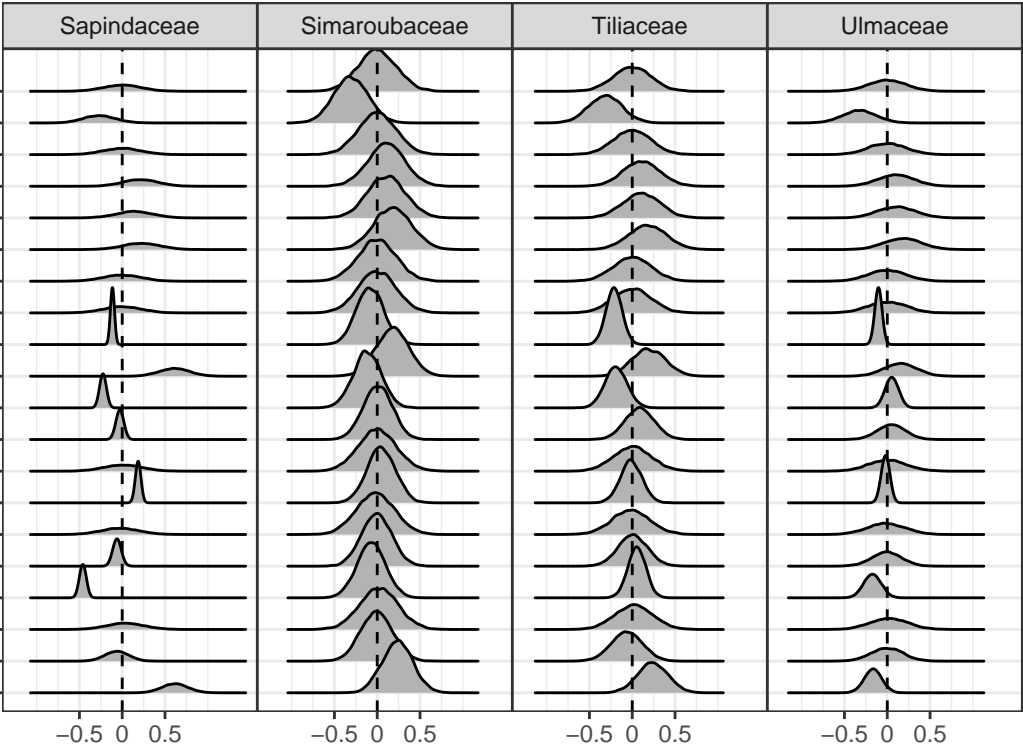

-0.5 0 0.5

-0.5 0 0.5

-0.5 0 0.5

-0.5 0 0.5

$\lambda$

Supplement: S1 Fig — Read across rows for that family’s competitive effect on other families and down columns for how a family responses to the competition of other families. (PDF) [file pone.0229930.s001.pdf]

A

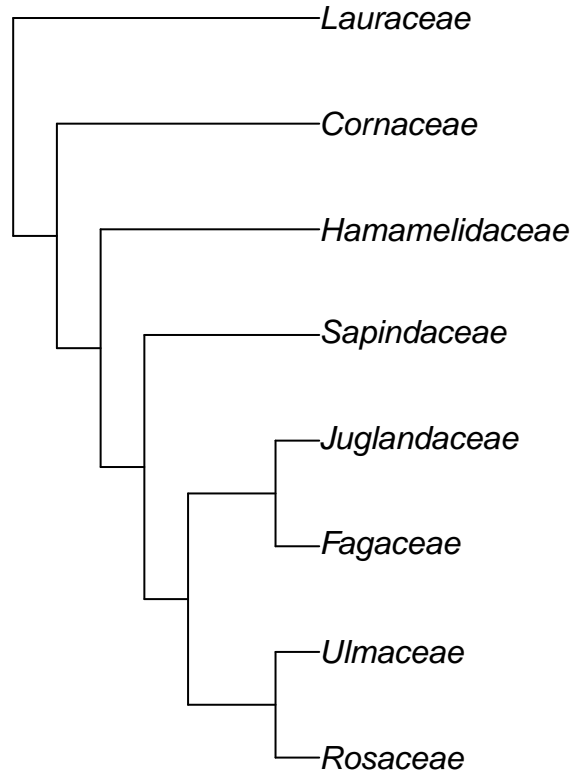

B

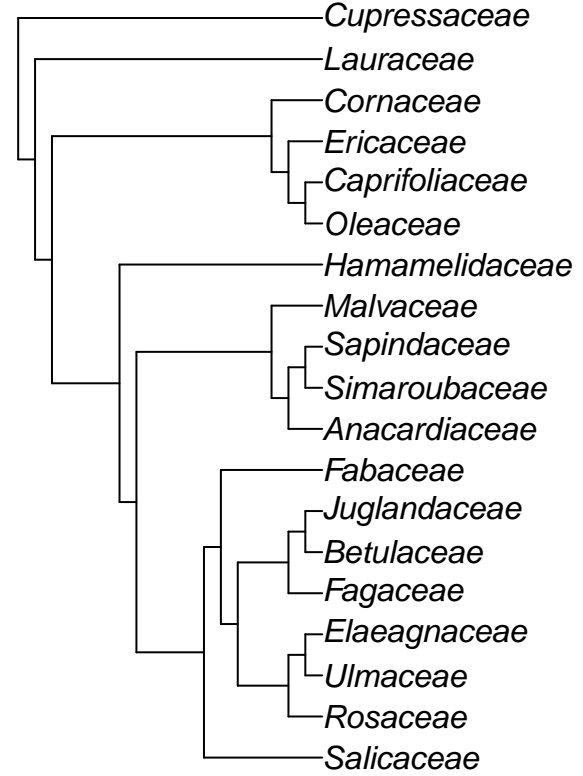

Supplement: S2 Fig — The phylogenetic relationship of families pulled from the Open Tree of Life [31] using the R package rotl [32]. A) The phylogeny for the most common families, this corresponds to the families shown in Fig 5. B) The phylogeny for all families in the plot, this corresponds to families shown in S1 Fig. (PDF) [file pone.0229930.s002.pdf]
