# Supplementary material for: A permutation test and spatial cross-validation approach to assess models of interspecific competition between trees
Source: PLoS One. 2020 Mar 11;15(3):e0229930. doi: 10.1371/journal.pone.0229930 (PMC7065802; doi:10.1371/journal.pone.0229930)
Supplement: S3 Fig — The total width of each horizontal bar represents the total number of neighbors (or competitors) of focal trees of a particular family in the study region. Within each bar, the width of each color represents the total number of competitor trees of a particular family within a neighborhood of 7.5 meters of trees of the focal family. For clarity this figure shows counts for the eight families with at least 200 individuals in the plot. This figure provides sample sizes for Fig 5. (PDF) [file pone.0229930.s003.pdf]

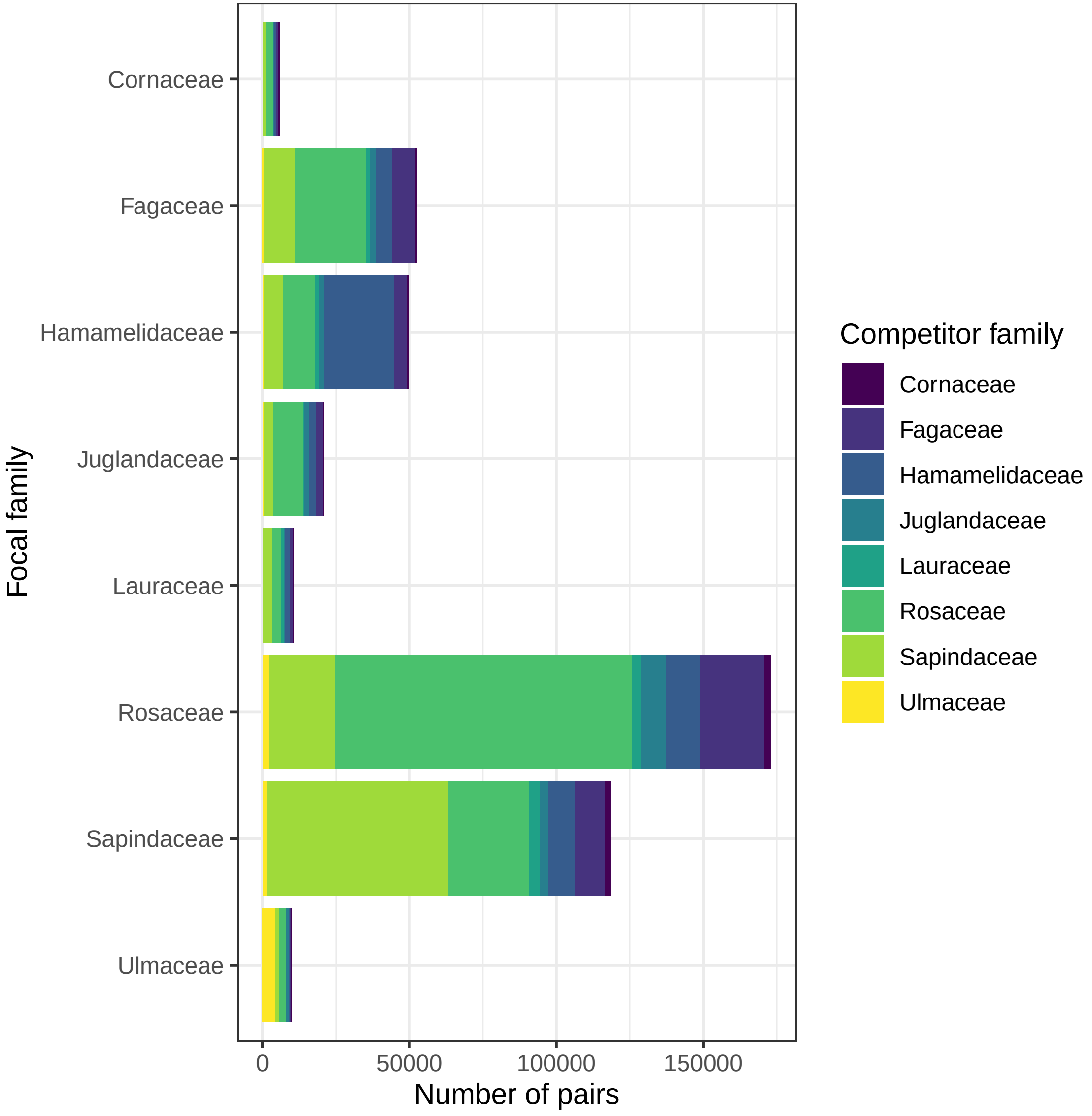

Focal family
Cornaceae
Fagaceae
Hamamelidaceae
Juglandaceae
Lauraceae
Rosaceae
Sapindaceae
Ulmaceae
0
50000
100000
150000
Number of pairs
Competitor family
Cornaceae
Fagaceae
Hamamelidaceae
Juglandaceae
Lauraceae
Rosaceae
Sapindaceae
Ulmaceae
