# Supplementary material for: A permutation test and spatial cross-validation approach to assess models of interspecific competition between trees
Source: PLoS One. 2020 Mar 11;15(3):e0229930. doi: 10.1371/journal.pone.0229930 (PMC7065802; doi:10.1371/journal.pone.0229930)
Supplement: S1 Appendix — (PDF) [file pone.0229930.s004.pdf]

Supporting information

S1 Appendix. Closed-form solutions for Bayesian Linear Regression.
Consider a standard linear model $y_i = \beta_0 + \beta_1 x_{i,1} + \ldots + \beta_p x_{i,p} + \epsilon_i$ for $i = 1, \ldots, n$ expressed in matrix form:

$$\boldsymbol{y} = X\boldsymbol{\beta} + \boldsymbol{\epsilon} \tag{9}$$

where

- $\boldsymbol{y} = [y_i]_{i=1}^n$ is the outcome variable vector of length n.
- $X = [\boldsymbol{x}_i^T]_{i=1}^n$ is the model matrix of dimension $n \times (p+1)$ where we have a column of 1's for the intercept and p covariates.
- $\boldsymbol{\beta} = [\beta_j]_{j=0}^p$ is the population parameter vector of regression coefficients of length $(p+1)$.
- $\boldsymbol{\epsilon} = [\epsilon_i]_{i=1}^n \sim MVN(\boldsymbol{0}, \sigma^2 I_n)$ is the vector of random error terms, where σ^2 is an unknown variance parameter.

thus we have a total of $(p+1)+1 = p+2$ parameters of interest.
Normal/Inverse Gamma (NIG) conjugacy: The analytic/closed-form solution to the posterior distribution of all $p+2$ parameters of interest from the model above exploits Normal/Inverse Gamma (NIG) conjugacy of the following 4 parameters:

- $\boldsymbol{\mu}$ a mean hyperparameter vector for $\boldsymbol{\beta}$ of length $(p+1)$.
- V a covariance hyperparameter matrix for $\boldsymbol{\beta}$ of dimension $(p+1) \times (p+1)$.
- a a shape hyperparemeter for σ^2 which is a scalar > 0.
- b a scale hyperparemeter for σ^2 which is a scalar > 0.

Prior distribution: After specifying prior hyperparameter values for $\boldsymbol{\mu}_0$, V_0, $a_0 > 0$, and $b_0 > 0$ we have:

$$\begin{aligned}
p(\boldsymbol{\beta}, \sigma^2) &= \text{NIG}(\boldsymbol{\mu}_0, V_0, a_0, b_0) && (10)\\
&= N(\boldsymbol{\mu}_0, \sigma^2 V_0) \times IG(a_0, b_0) && (11)\\
&= p(\boldsymbol{\beta} \left| \sigma^2 \right.) \times p(\sigma^2) && (12)
\end{aligned}$$

where

$$\begin{aligned}
p(\sigma^2) &= \frac{b_0^{a_0}}{\Gamma(a_0)} \left(\frac{1}{\sigma^2}\right)^{a_0+1} \exp\left(-\frac{b_0}{\sigma^2}\right) && (13)\\
&= \text{Inverse-Gamma}(a_0, b_0) && (14)
\end{aligned}$$

and

$$\begin{aligned}
p(\boldsymbol{\beta}) &= \int_0^\infty p(\boldsymbol{\beta} \left| \sigma^2 \right.) \times p(\sigma^2) d\sigma^2 && (15)\\
&= \frac{\Gamma\left(\frac{\nu_0+p}{2}\right)}{\Gamma\left(\frac{\nu_0}{2}\right) \pi^{p/2} \left|\nu_0 \Sigma\right|^{1/2}} \left[1 + \frac{(\boldsymbol{\beta}-\boldsymbol{\mu}_0)^T \Sigma^{-1} (\boldsymbol{\beta}-\boldsymbol{\mu}_0)}{\nu_0}\right]^{-\frac{\nu_0+p}{2}} && (16)\\
&= \text{Multivariate } t_{df=\nu_0}(\boldsymbol{\mu}_0, \Sigma_0) \text{ for } \nu_0 = 2a_0 \text{ and } \Sigma_0 = \frac{b_0}{a_0} V_0 && (17)
\end{aligned}$$

Posterior distribution: Thus given the likelihood $p(\boldsymbol{y}|\boldsymbol{\beta}, \sigma^2) = \text{MVN}(X\boldsymbol{\beta}, \sigma^2 I)$, we have

$$
\begin{aligned}
p(\boldsymbol{\beta}, \sigma^2|\boldsymbol{y}) &= \frac{p(\boldsymbol{y}|\boldsymbol{\beta}, \sigma^2)p(\boldsymbol{\beta}, \sigma^2)}{p(\boldsymbol{y})} &(18)\\
&= \text{NIG}(\boldsymbol{\mu}^*, V^*, a^*, b^*) &(19)\\
p(\sigma^2|\boldsymbol{y}) &= \text{Inverse-Gamma}(a^*, b^*) &(20)\\
p(\boldsymbol{\beta}|\boldsymbol{y}) &= \text{Multivariate } t_{df=\nu^*}(\boldsymbol{\mu}^*, \Sigma^*) \text{ for } \nu^* = 2a^* \text{ and } \Sigma^* = \frac{b^*}{a^*}V^* &(21)
\end{aligned}
$$

with posterior hyperparameter values

$$
\begin{aligned}
\boldsymbol{\mu}^* &= (V_0^{-1} + X^T X)^{-1}(V_0^{-1}\boldsymbol{\mu}_0 + X^T \boldsymbol{y}) &(22)\\
V^* &= (V_0^{-1} + X^T X)^{-1} &(23)\\
a^* &= a_0 + \frac{n}{2} &(24)\\
b^* &= b_0 + \frac{1}{2}\left[\boldsymbol{\mu}_0^T V_0^{-1}\boldsymbol{\mu}_0 + \boldsymbol{y}^T\boldsymbol{y} - \boldsymbol{\mu}^{*T} V^{*-1}\boldsymbol{\mu}^*\right] &(25)
\end{aligned}
$$

Posterior predictive distribution: In a Bayesian framework, given a set of observed outcome variables $\boldsymbol{y}$ the posterior predictive distribution of a new observations $\tilde{\boldsymbol{y}}$ is [22]:

$$
p\left(\tilde{\boldsymbol{y}}\,|\boldsymbol{y}\right) = \int_{\boldsymbol{\Theta}} p\left(\tilde{\boldsymbol{y}}, \boldsymbol{\Theta}\,|\boldsymbol{y}\right) d\boldsymbol{\Theta} = \int_{\boldsymbol{\Theta}} p\left(\tilde{\boldsymbol{y}}\,|\boldsymbol{\Theta}, \boldsymbol{y}\right) \times p\left(\boldsymbol{\Theta}\,|\boldsymbol{y}\right) d\boldsymbol{\Theta} \qquad (26)
$$

While a frequentist approach would use $p(\tilde{\boldsymbol{y}}|\widehat{\boldsymbol{\Theta}}, \boldsymbol{y})$ based on the maximum likelihood estimate vector $\widehat{\boldsymbol{\Theta}}$, the above Bayesian posterior formulation accounts for the uncertainty about $\boldsymbol{\Theta}$ by integrating $p\left(\tilde{\boldsymbol{y}}\,|\boldsymbol{\Theta}, \boldsymbol{y}\right)$ over the posterior distribution $p\left(\boldsymbol{\Theta}\,|\boldsymbol{y}\right)$. Hence, the posterior predictive distribution will have higher variance.

In the case of our Bayesian linear regression model, we have $\boldsymbol{\Theta} = \left\{\boldsymbol{\beta}, \sigma^2\right\}$. For a new model matrix $\tilde{X}$ of dimension $m \times (p+1)$ based on m new observations we'd like to make a prediction $\tilde{\boldsymbol{y}}$ for:

$$
\begin{aligned}
p(\tilde{\boldsymbol{y}}|\boldsymbol{y}) &= \int p(\tilde{\boldsymbol{y}}, \boldsymbol{\beta}, \sigma^2|\boldsymbol{y}) d\boldsymbol{\beta} d\sigma^2 &(27)\\
&= \int p(\tilde{\boldsymbol{y}}|\boldsymbol{\beta}, \sigma^2, \boldsymbol{y}) \times p(\boldsymbol{\beta}, \sigma^2|\boldsymbol{y}) d\boldsymbol{\beta} d\sigma^2 &(28)\\
&= \int MVN(\tilde{X}\boldsymbol{\beta}, \sigma^2 I) \times \text{NIG}(\boldsymbol{\mu}^*, V^*, a^*, b^*) d\boldsymbol{\beta} d\sigma^2 &(29)\\
&= \text{Multivariate } t_{df=\nu^*}\left(\tilde{X}\boldsymbol{\mu}^*, \frac{b^*}{a^*}(I + \tilde{X}V^*\tilde{X}^T)\right) &(30)
\end{aligned}
$$
