# Supplementary material for: A permutation test and spatial cross-validation approach to assess models of interspecific competition between trees
Source: PLoS One. 2020 Mar 11;15(3):e0229930. doi: 10.1371/journal.pone.0229930 (PMC7065802; doi:10.1371/journal.pone.0229930)
Supplement: S2 Appendix — (PDF) [file pone.0229930.s005.pdf]

S2 Appendix Species list.

Common name	Scientific name	Family
Alternate-leaf dogwood	*Cornus alternifolia*	Cornaceae
American basswood	*Tilia americana*	Tiliaceae
American beech	*Fagus grandifolia*	Fagaceae
American elm	*Ulmus americana*	Ulmaceae
Autumn olive	*Elaeagnus umbellata*	Elaeagnaceae
Big tooth aspen	*Populus grandidentata*	Salicaceae
Bitternut hickory	*Carya cordiformis*	Juglandaceae
Black cherry	*Prunus serotina*	Rosaceae
Black locust	*Robinia pseudoacacia*	Fabaceae
Black oak	*Quercus velutina*	Fagaceae
Black walnut	*Juglans nigra*	Juglandaceae
Black/northern pin oak hybrid	*Quercus velutina x ellipsoidalis*	Fagaceae
Black/red oak hybrid	*Quercus velutina x rubra*	Fagaceae
Choke cherry	*Prunus virginiana*	Rosaceae
Elderberry	*Sambucus canadensis*	Adoxaceae
Flowering dogwood	*Cornus florida*	Cornaceae
Gray dogwood	*Cornus racemosa*	Cornaceae
Hawthorn	*Crataegus* sp	Rosaceae
Hazelnut	*Corylus americana*	Betulaceae
Hophornbeam	*Ostrya virginiana*	Betulaceae
Juniper	*Juniperus communis*	Cupressaceae
Musclewood	*Carpinus caroliniana*	Betulaceae
Northern white cedar	*Thuja occidentalis*	Cupressaceae
Pignut hickory	*Carya glabra*	Juglandaceae
Prickly gooseberry	*Ribes cynosbati*	Grossulariaceae
Prickly ash	*Zanthoxylum americanum*	Rutaceae
Red maple	*Acer rubrum*	Sapindaceae
Red oak	*Quercus rubra*	Fagaceae
Sassafras	*Sassafras albidum*	Lauraceae
Serviceberry	*Amelanchier arborea*	Rosaceae
Shagbark hickory	*Carya ovata*	Juglandaceae
Silky dogwood	*Cornus amomum*	Cornaceae
Spicebush	*Lindera benzoin*	Lauraceae
Sugar maple	*Acer saccharum*	Sapindaceae
Tree of heaven	*Ailanthus altissima*	Simaroubaceae
White ash	*Fraxinus americana*	Oleaceae
White oak	*Quercus alba*	Fagaceae
Witch hazel	*Hamamelis virginiana*	Hamamelidaceae
Yellow birch	*Betula alleghaniensis*	Betulaceae
Eastern red cedar	*Juniperus virginiana*	Cupressaceae
Northern pin oak	*Quercus ellipsoidalis*	Fagaceae
