# Supplementary material for: A permutation test and spatial cross-validation approach to assess models of interspecific competition between trees
Source: PLoS One. 2020 Mar 11;15(3):e0229930. doi: 10.1371/journal.pone.0229930 (PMC7065802; doi:10.1371/journal.pone.0229930)
Supplement: Attachment — Submitted filename: PONE-D-19-24831_reviewer.pdf [file pone.0229930.s006.pdf]

PLOS ONE

A permutation test and spatial cross-validation approach to assess models of interspecies competition between trees

--Manuscript Draft--

**Manuscript Number:**	PONE-D-19-24831
**Article Type:**	Research Article
**Full Title:**	A permutation test and spatial cross-validation approach to assess models of interspecies competition between trees
**Short Title:**	Permutation test and spatial cross-validation approach to assess models of competition
**Corresponding Author:**	David Allen<br>Middlebury College<br>Middlebury, Vermont UNITED STATES
**Keywords:**	interspecies competition; spatial cross validation; oak-hickory forest
**Abstract:**	Measuring species-specific competitive interactions is key to understanding plant communities. Repeat censused large forest dynamics plots offer an ideal setting to measure these interactions by estimating the species-specific competitive effect on neighboring tree growth. Estimating these interaction values can be difficult because the number of interactions grows with the square of the number of species. Furthermore, confidence in the estimates can be overestimated if any spatial structure of errors is not considered. Here we measure these interactions in a forest dynamics plot in a transitional oak-hickory forest. We analytically fit Bayesian linear regression models of annual tree radial growth as a function of that tree's species, its size, and its neighboring trees. We then compare these models to test whether the identity of a tree's neighbors matters and if so at what level: based on trait grouping, based on phylogenetic family, or based on species. We use a spatial cross-validation scheme to better estimate model errors while avoiding potentially over-fitting our model. Since our model is analytically solvable we can rapidly evaluate it, which allows our proposed cross-validation scheme to be computationally feasible. We find that the identity of the focal and competitor trees matter for competitive interactions, but, surprisingly identity matters at the family rather than species level.
**Order of Authors:**	David Allen
	Albert Kim
**Additional Information:**	
**Question**	**Response**
**Financial Disclosure**<br><br>Enter a financial disclosure statement that describes the sources of funding for the work included in this submission. Review the submission guidelines for detailed requirements. View published research articles from *PLOS ONE* for specific examples.<br><br>This statement is required for submission and **will appear in the published article** if the submission is accepted. Please make sure it is accurate.	This work was supported by the Edwin S. George Reserve Fund and by a USDA McIntyre-Stennis Grant. The funders had no role in study design, data collection and analysis, decision to publish, or preparation of the manuscript.

Powered by Editorial Manager® and ProduXion Manager® from Aries Systems Corporation

**Unfunded studies**<br>Enter: *The author(s) received no specific funding for this work.*<br><br>**Funded studies**<br>Enter a statement with the following details:<br>• Initials of the authors who received each award<br>• Grant numbers awarded to each author<br>• The full name of each funder<br>• URL of each funder website<br>• Did the sponsors or funders play any role in the study design, data collection and analysis, decision to publish, or preparation of the manuscript?<br>• **NO** - Include this sentence at the end of your statement: *The funders had no role in study design, data collection and analysis, decision to publish, or preparation of the manuscript.*<br>• **YES** - Specify the role(s) played.<br><br>* typeset	
**Competing Interests**<br><br>Use the instructions below to enter a competing interest statement for this submission. On behalf of all authors, disclose any competing interests that could be perceived to bias this work—acknowledging all financial support and any other relevant financial or non-financial competing interests.<br><br>This statement **will appear in the published article** if the submission is accepted. Please make sure it is accurate. View published research articles from *PLOS ONE* for specific examples.	The authors have declared that no competing interests exist.

**NO authors have competing interests**<br><br>Enter: *The authors have declared that no competing interests exist.*<br><br>**Authors with competing interests**<br><br>Enter competing interest details beginning with this statement:<br><br>*I have read the journal's policy and the authors of this manuscript have the following competing interests: [insert competing interests here]*<br><br>* typeset	
**Ethics Statement**<br><br>Enter an ethics statement for this submission. This statement is required if the study involved:<br><br>• Human participants<br>• Human specimens or tissue<br>• Vertebrate animals or cephalopods<br>• Vertebrate embryos or tissues<br>• Field research<br><br>Write "N/A" if the submission does not require an ethics statement.<br><br>General guidance is provided below. Consult the submission guidelines for detailed instructions. **Make sure that all information entered here is included in the Methods section of the manuscript.**	N/A

Powered by Editorial Manager® and ProduXion Manager® from Aries Systems Corporation

**Format for specific study types**<br><br>**Human Subject Research (involving human participants and/or tissue)**<br>• Give the name of the institutional review board or ethics committee that approved the study<br>• Include the approval number and/or a statement indicating approval of this research<br>• Indicate the form of consent obtained (written/oral) or the reason that consent was not obtained (e.g. the data were analyzed anonymously)<br><br>**Animal Research (involving vertebrate animals, embryos or tissues)**<br>• Provide the name of the Institutional Animal Care and Use Committee (IACUC) or other relevant ethics board that reviewed the study protocol, and indicate whether they approved this research or granted a formal waiver of ethical approval<br>• Include an approval number if one was obtained<br>• If the study involved *non-human primates*, add *additional details* about animal welfare and steps taken to ameliorate suffering<br>• If anesthesia, euthanasia, or any kind of animal sacrifice is part of the study, include briefly which substances and/or methods were applied<br><br>**Field Research**<br><br>Include the following details if this study involves the collection of plant, animal, or other materials from a natural setting:<br>• Field permit number<br>• Name of the institution or relevant body that granted permission	
**Data Availability**<br><br>Authors are required to make all data underlying the findings described fully available, without restriction, and from the time of publication. PLOS allows rare exceptions to address legal and ethical concerns. See the PLOS Data Policy and FAQ for detailed information.	Yes - all data are fully available without restriction

A Data Availability Statement describing where the data can be found is required at submission. Your answers to this question constitute the Data Availability Statement and **will be published in the article**, if accepted.<br><br>**Important:** Stating 'data available on request from the author' is not sufficient. If your data are only available upon request, select 'No' for the first question and explain your exceptional situation in the text box.<br><br>Do the authors confirm that all data underlying the findings described in their manuscript are fully available without restriction?	
**Describe where the data may be found in full sentences. If you are copying our sample text, replace any instances of XXX with the appropriate details.**<br><br>• If the data are **held or will be held in a public repository**, include URLs, accession numbers or DOIs. If this information will only be available after acceptance, indicate this by ticking the box below. For example: *All XXX files are available from the XXX database (accession number(s) XXX, XXX.)*.<br>• If the data are all contained **within the manuscript and/or Supporting Information files**, enter the following: *All relevant data are within the manuscript and its Supporting Information files.*<br>• If neither of these applies but you are able to provide **details of access elsewhere**, with or without limitations, please do so. For example:<br><br>*Data cannot be shared publicly because of [XXX]. Data are available from the XXX Institutional Data Access / Ethics Committee (contact via XXX) for researchers who meet the criteria for access to confidential data.*<br><br>*The data underlying the results presented in the study are available from (include the name of the third party*	The data will be deposited in Deep Blue Data shortly. This is a University of Michigan data repository.<br><br>The data will have a DOI.<br>https://deepblue.lib.umich.edu/data

and contact information or URL).<br>• This text is appropriate if the data are owned by a third party and authors do not have permission to share the data.<br><br>* typeset	
Additional data availability information:	

Manuscript Click here to download Manuscript paper_noSI.pdf

A permutation test and spatial cross-validation approach to assess models of interspecies competition between trees

David Allen[1◑¤*], Albert Y. Kim[2◑]

1 Department of Biology, Middlebury College, Middlebury, VT, USA
2 Statistical and Data Sciences Program, Smith College, Northampton, MA, USA

◑These authors contributed equally to this work.
¤Current Address: Department of Biology, Middlebury College, Middlebury, VT, USA
* dallen@middlebury.edu

Abstract

Measuring species-specific competitive interactions is key to understanding plant communities. Repeat censused large forest dynamics plots offer an ideal setting to measure these interactions by estimating the species-specific competitive effect on neighboring tree growth. Estimating these interaction values can be difficult because the number of interactions grows with the square of the number of species. Furthermore, confidence in the estimates can be overestimated if any spatial structure of errors is not considered. Here we measure these interactions in a forest dynamics plot in a transitional oak-hickory forest. We analytically fit Bayesian linear regression models of annual tree radial growth as a function of that tree's species, its size, and its neighboring trees. We then compare these models to test whether the identity of a tree's neighbors matters and if so at what level: based on trait grouping, based on phylogenetic family, or based on species. We use a spatial cross-validation scheme to better estimate model errors while avoiding potentially over-fitting our model. Since our model is analytically solvable we can rapidly evaluate it, which allows our proposed cross-validation scheme to be computationally feasible. We find that the identity of the focal and competitor trees matter for competitive interactions, but, surprisingly identity matters at the family rather than species level.

Introduction

Competition is a key biotic interaction which structures communities. To better understand the role it plays we need ways to understand species-specific competitive interactions. Attempts have been made to do this through direct measurement of species-specific competition coefficients, or to generalize competitive interactions based on trait or phylogeny differences between competitors.

Repeat censused forest inventory plots are good places to measure species-specific competition. In such forests trees are identified, mapped, and have their diameter measured at regular intervals [1,2]. Diameter growth between censuses of individual trees is modeled as a function of neighborhood tree species identity, size, and distance to focal tree [3,4]. This gives a way to measure the species-specific effect of competition on tree growth.

The first question is whether species identity of the competitor matters at all [5], or is competition neutral (sensu [6]). Assuming that the identity of competitors does

matter then it can be hard to measure all of the interaction coefficients, which increase proportionally with the square of species number in the community. Furthermore, some species pairs might not coexist nearby one another enough to get any data to measure their effect on one another. A number of approaches have been taken to deal with this.

In low-diversity forests attempts have been made to measure species-specific competition coefficients for the effect of competition on radial tree growth [7–10]. In a western US, evergreen forest, Das [8] was able to estimate the 16 competition coefficients among the four dominant tree species. Canham et al. [7] measured the competition coefficients between the 14 most common tree species in Northern New England. While they were able to estimate most coefficients, for species-pairs that rarely occurred together ~~they could not.~~

A hierarchical Bayesian approach can be take to address this [11]. All species-pairs are considered, no data are thrown out, and competition coefficients are "regressed back" to an overall average. The amount of regression decreases with number of interacting pairs of individuals of that species pair. This approach was taken by Tatsumi et al. [9] when looking at 38 tree species in a cool-temperate mixed conifer-broadleaf forest. They found support for the hierarchical model over non-hierarchical version, but because of relatively small sample size, competition coefficients of only a few species pairs had 95% credible intervals that did not overlap with the hyperparameter.

A final approach is to estimate competition coefficients based on species attributes, rather than measure each one. A common approach is to correlate competition coefficients with either trait or phylogenetic distance [12]. Uriarte et al. [12] found that trait distance did a better job of explaining competitive interactions than phylogenetic distance. Kunstler et al. [13] also find that traits can explain competitive interactions.

For any of these approaches a method for comparing model performance is needed. Previous studies have used information criteria to compare models [7–9, 12]. Such information criteria are asymptotic approximations for out-of-sample-prediction error [14]. Another commonly used measure of out-of-sample-prediction error is the more easily interpretable mean squared error [15].

Here we present a method to answer two questions from repeat censused forest inventory plots: (1) "Does the species identity of neighboring competitor trees matter?" and (2) "If so how can you measure species-specific competition coefficients?" For both questions we analytically derived posterior estimates of coefficients of a linear Bayesian neighborhood competition model. The chief advantage of this analytic approach is that the aforementioned coefficients are much less computationally expensive to estimate than other approaches, such as the Markov chain Monte Carlo based estimates necessitated by other more complicated models [7, 9].

To answer question (1), we use a permutation test based approach. We first fit the model and obtain an observed test statistic assessing the quality of the model fit, in our case the observed root mean square error (RMSE). We then permute the competitor species identities and compute a permuted test statistic a large number of times, thereby empirically generating a "null" distribution of the test statistic. We then compare the observed test statistic to this null distribution to obtain significance measures. The rapid analytic solution is required for this permutation test to run in a reasonable amount of time. We find that identity of the competitor species does matter.

For question (2), we can further ask whether species identity, family identity, or trait values best explains competitive interactions. Here we provide a method to compare spatially cross-validated RMSEs of competing models. The rapid analytic solution is again required to fit the model repeatedly in the cross-validation scheme. Surprisingly we find that grouping species by family best explains competitive interactions.

Materials and methods

Study site and sampling

The Big Woods plot is a 23 ha forest dynamics plot in Pickney, MI (42.462902 N, 84.006093 W). The plot is within a transitional oak-hickory forest. The canopy is dominated by black oak, northern red oak, white oak, bitternut hickory, and shagbark hickory. But there are relatively few oaks in the mid and understory, instead these strata are dominated by red maple and black cherry. The elevation in the plot ranges from 270 m to 305 m. Above 275 m the mineral soils are largely Boyer–Oshtemo sandy loam, and below 275 m the soils are mainly histols dominated by Carlisle and Rifle muck. The rugged topography within the plot is the result of glacial scouring with hills and knobs separated by kettle holes and basins. For more information on the plot see Allen et al. [16].

The original plot was 12 ha, in this plot all free-standing woody stems larger than 3.2 cm diameter at breast height (DBH) were censused, had their DBH measured, mapped, and identified. Between 2007 and 2010 this plot was expanded to 23 ha using the same censusing technique and the original 12 ha were re-censused. In 2014 the entire 23 ha plot was re-censused to determine the diameter growth of each individual, tag individuals recruited into the greater than 3.2 cm DBH size class, and identify individuals which died. For this analysis we consider the average annual diameter growth between the 2007–2010 and 2014 censuses for all stems alive in both censuses ignoring resprouts. We define stems alive in the 2007–2010 census as the competitor stems.

The plot is part of the Smithsonian Institution's ForestGEO global network of forest research plots (Smithsonian Institution, Washington DC, USA).

Species grouping methods

We tested which grouping of trees best explained competitive interactions. We grouped species in three ways: (1) by species, (2) by family, and (3) according to traits assumed to correlate with competitive interactions. To form this third grouping, we collected species trait values from the TRY database [17]. From this we picked plant height, wood density, and specific leaf area as our traits of interest. ~~These traits~~ have been identified as important to tree competition [13]. We clustered these species based on their values for these three traits using the `cluster` package in R [18, 19]. The distance matrix was formed by the euclidean distance between species' three trait values. Hierarchical clustering of this distance matrix using `agnes` command. We chose to cut the trees into six groups.

Neighborhood-effect growth model

Let $i = 1, \ldots, n_j$ index all n_j trees of "focal" species group j; let $j = 1, \ldots, J$ index all J focal species groups; and let $k = 1, \ldots, K$ index all K "competitor" species groups. We model the growth in diameter per year y_{ij} (in centimeters) of the ith tree of focal species group j as a linear model f of the following covariates $\vec{x}_{ij}$

$$y_{ij} = f(\vec{x}_{ij}) + \epsilon_{ij} = \beta_{0,j} + \beta_{\text{dbh},j} \cdot \text{dbh}_{ij} + \sum_{k=1}^{K} \lambda_{jk} \cdot \text{BA}_{ijk} + \epsilon_{ij} \tag{1}$$

where dbh_{ij} is the diameter at breast height (in centimeters) of the focal tree, BA_{ijk} is sum of the basal area of all trees of competitor species group k within a neighborhood of 7.5 meters of the focal tree, and ϵ_{ij} is a random error term distributed Normal$(0, \sigma^2)$.

We will consider models where the focal species grouping $j = 1, \ldots, J$ and the competitor species grouping $k = 1, \ldots, K$ will reflect the three notions of species grouping introduced earlier: trait group with 6 groups, phylogenetic family with 20 groups, and actual species with 36 groups. While our model specification is flexible enough where our notion of focal tree grouping does not necessarily have to match our notion of competitor tree grouping, for simplicity in this paper we will only consider models where both notions match and hence $J = K$.

Furthermore, our models will incorporate a specific notion of competition reflecting a particular assumption on the nature of competition between trees: *species grouping-specific effects of competition*. For a given focal species group j, all competitor species groups exert different competitive effects. Such models not only assume competition between trees exists, but also that different focal versus competitor species group pairs have different competitive relationships.

More specifically, using the above three notions of species grouping, we'll compare three different models for growth y_{ij}, each with different numbers of $(\boldsymbol{\beta}_0, \boldsymbol{\beta}_{\text{dbh}}, \boldsymbol{\lambda}, \sigma^2)$ parameters to estimate.

1. Trait group with $J = K = 6$, thus $(\boldsymbol{\beta}_0, \boldsymbol{\beta}_{\text{dbh}}, \sigma^2)$ consist of 6 + 6 + 1 = 13 parameters. Furthermore, there are 6 × 6 unique values of λ_{jk}, thus $\boldsymbol{\lambda}$ is a 6×6 matrix, totaling 13 + 6 × 6 = 49 parameters.

2. Phylogenetic family with $J = K = 20$, thus $(\boldsymbol{\beta}_0, \boldsymbol{\beta}_{\text{dbh}}, \sigma^2)$ consist of 20 + 20 + 1 = 41 parameters. Furthermore, there are 20 × 20 unique values of λ_{jk}, thus $\boldsymbol{\lambda}$ is a 20×20 matrix, totaling 41 + 20 × 20 = 441 parameters.

3. Actual species with $J = K = 36$, thus $(\boldsymbol{\beta}_0, \boldsymbol{\beta}_{\text{dbh}}, \sigma^2)$ consist of 36 + 36 + 1 = 73 parameters. Furthermore, there are 36 × 36 unique values of λ_{jk}, thus $\boldsymbol{\lambda}$ is a 36×36 matrix, totaling 73 + 36 × 36 = 1369 parameters.

For each of these three models, all $(\boldsymbol{\beta}_0, \boldsymbol{\beta}_{\text{dbh}}, \boldsymbol{\lambda}, \sigma^2)$ parameter estimates are obtained via Bayesian linear regression. We favor a Bayesian approach since they allow us to incorporate prior information about all the parameters in Eq (1) [20]. This in turn serves us when particular species grouping pairs are rare, leading to posterior distributions that are more weighted towards the prior than the likelihood [20]. While the linear regression model in Eq (1) is much less complex than the model formulations considered by [7], both the posterior distribution and posterior predictive distribution of all parameters have analytic and closed-form solutions. Thus we are saved from the computational expense of using methods to approximate the posterior such as Markov chain Monte Carlo [21], [22]. These savings in computational expense will be important given the large number of times ~~we'll~~ fit the model in Eq (1), as we will outline in the upcoming sections on the permutation test and spatial cross-validation we used.

We present a brief summary of the closed form solutions to Bayesian linear regression here, leaving fuller detail in S1 Appendix. For simplicity of notation, let $\boldsymbol{\beta}$ represent the parameters $\boldsymbol{\beta}_0, \boldsymbol{\beta}_{\text{dbh}}, \boldsymbol{\lambda}$. The likelihood function $p(\boldsymbol{y}|\boldsymbol{\beta}, \sigma^2)$ of our observed growths resulting from Eq (1) is Multivariate Normal$(X\boldsymbol{\beta}, \sigma^2 I_n)$ where I_n is the $n \times n$ identity matrix. Bayesian linear regression exploits the fact that the Multivariate Normal-inverse-Gamma distribution is a conjugate prior of the Multivariate Normal distribution. So given our Multivariate Normal likelihood $p(\boldsymbol{y}|\boldsymbol{\beta}, \sigma^2)$, by assuming that the joint prior distribution $\pi(\boldsymbol{\beta}, \sigma^2)$ of $\boldsymbol{\beta}, \boldsymbol{\sigma^2}$ is NIG$(\boldsymbol{\mu}_0, V_0, a_0, b_0)$ with the following hyperparameters: 1) a mean vector $\boldsymbol{\mu}_0$ for $\boldsymbol{\beta}$, 2) a shape matrix V_0 for $\boldsymbol{\beta}$, 3) shape $a_0 > 0$ for σ^2, and scale $b_0 > 0$ for σ^2, the joint posterior distribution $\pi(\boldsymbol{\beta}, \sigma^2|\boldsymbol{y})$ is also NIG$(\boldsymbol{\mu}^*, V^*, a^*, b^*)$ with:

$$\begin{aligned}
\boldsymbol{\mu}^* &= (V_0^{-1} + X^T X)^{-1}(V_0^{-1}\boldsymbol{\mu}_0 + X^T\boldsymbol{y}) & (2)\\
V^* &= (V_0^{-1} + X^T X)^{-1} & (3)\\
a^* &= a_0 + \frac{n}{2} & (4)\\
b^* &= b_0 + \frac{1}{2}\left[\boldsymbol{\mu}_0^T V_0^{-1}\boldsymbol{\mu}_0 + \boldsymbol{y}^T\boldsymbol{y} - \boldsymbol{\mu}^{*T}V^{*-1}\boldsymbol{\mu}^*\right] & (5)
\end{aligned}$$

It can be shown that the marginal prior distribution $\pi(\sigma)$ is Inverse-Gamma(a_0, b_0) while $\pi(\boldsymbol{\beta})$ is Multivariate-t with location vector $\boldsymbol{\mu}_0$, shape matrix $\Sigma_0 = \frac{b_0}{a_0}V_0$, and degrees of freedom $\nu_0 = 2a_0$. Given the aforementioned prior conjugacy, the marginal posterior distribution $\pi(\sigma|\boldsymbol{y})$ is also Inverse-Gamma while $\pi(\boldsymbol{\beta}|\boldsymbol{y})$ is also Multivariate-t, both with updated $\boldsymbol{\mu}^*, V^*, a^*, b^*$ hyperparameter values in place of $\boldsymbol{\mu}_0, V_0, a_0, b_0$.

Furthermore, it can also be shown that the posterior predictive distribution $p(\tilde{\boldsymbol{y}}|\boldsymbol{y})$ for a model matrix $\tilde{X}$ corresponding to a new set of observed covariates is Multivariate-t with location vector $\tilde{X}\boldsymbol{\mu}^*$, shape matrix $\frac{b^*}{a^*}(I + \tilde{X}V^*\tilde{X}^T)$, and degrees of freedom $\nu^* = 2a^*$. The means of the posterior predictive distributions will be used to obtain fitted/predicted values $\widehat{y}_{ij}$.

Permutation test

Recall from the previous section our model assumes *species grouping-specific effects of competition*, whereby for a given focal species group j, all competitor species groups exert different competitive effects. The validity of this hypothesis can be evaluated using the following hypothesis test:

$$\begin{aligned}
& H_0 : \lambda_{jk} = \lambda_j \text{ for all } k = 1, \ldots, K & (6)\\
\text{vs.}\quad & H_A : \text{at least one } \lambda_{jk} \text{ is different} & (7)
\end{aligned}$$

whereby the null hypothesis H_0 reflects the hypothesized *no species grouping-specific effects of competition* while the alternative hypothesis H_A reflects the hypothesized *species grouping-specific effects of competition* of our three models.

We can therefore answer the question (1) from the introduction of whether the species identity of neighboring competitor trees matters using a permutation test (also called an "exact test"). We generate the null distribution of a test statistic of interest by randomly permuting the competitor species group labels k for all trees of focal species group j for a large number of iterations. Such permutations of the competitor species group labels (while holding all other variables constant) are permissible under the assumed null hypothesis above. After computing the test statistic for each iteration, we then compare this null distribution to the observed value of the test statistic to obtain measures of statistical significance. Given the large number of permutations and the corresponding large number of model fitting this will require, having the computationally inexpensive parameter estimates discussed above is all the more important.

Our test statistic will be a commonly-used and relatively simple measure of a model's predictive accuracy: the mean-squared error (RMSE) between all observed values $\boldsymbol{y}$ and all fitted/predicted values $\widehat{\boldsymbol{y}}$. Specifically, we'll compare all observed growths y_{ij} with their corresponding fitted/predicted growths $\widehat{y}_{ij}$ obtained from the posterior predictive distributions:

$$\text{RMSE}(\boldsymbol{y}, \widehat{\boldsymbol{y}}) = \sqrt{\frac{1}{n}\sum_{j=1}^{J}\sum_{i=1}^{n_j}\left(y_{ij} - \widehat{y}_{ij}\right)^2} \quad (8)$$

Fig 1. Big Woods study region with one iteration of spatial cross-validation algorithm. For this particular iteration of the cross-validation algorithm, we train our models on all 14 unnumbered blocks. We then apply the model to make predictions on block 10. Blocks 1, 2, 3, 9, 11, 13, 14, and 15 act as “buffer” blocks isolating the test block from the training blocks.

Spatial cross-validation

Among the most common methods for estimating out-of-sample predictive error is cross-validation, whereby independent “training” and “test” sets are created by resampling from the original sample of data. The model $\widehat{f}$ is first fit to the training set and then the model’s performance is evaluated on the test data [15]. However given the spatial structure of forest census data, there most likely exists spatial-auto-correlation between the individual trees and thus using individual trees as the resampling unit will violate the independence assumption inherent to cross-validation. One must instead resample spatial “blocks” of trees when creating training and test data, thereby preserving within block spatial-auto-correlations. Roberts et al. demonstrated that ignoring such spatial structure can lead to model error estimates that are overly optimistic and thus betray the true performance of any model’s predictive ability on new out-of-sample data [23].

To study the magnitude of this optimism, we’ll always report two sets or RMSE’s: one where both the model was fit and the RMSE evaluated on the same entire dataset and another where cross-validation is performed. On top of the large number of permutations, given the large number of iterations of model fits cross-validation will require, the importance of computationally inexpensive parameter estimates discussed earlier is ~~all the more~~ critical.

In Fig 1 we display the spatial distribution of the 27,192 trees in the Big Woods study region and illustrate one iteration of the spatial cross-validation algorithm. We superimpose a 100 $\times$ 100 meter grid onto the study region and then assign each of the 27,192 trees to one of 23 arbitrarily numbered spatial blocks. In this particular iteration of the algorithm, we first fit our model $\widehat{f}$ to all trees in the 14 unnumbered “training” blocks. We then apply the fitted model $\widehat{f}$ to all trees in the “test” block labeled 10 to obtain predicted growths $\widehat{y}$, which we then compare to observed growths y to obtain the estimated RMSE for this block. The remaining 8 labeled blocks 1, 2, 3, 9, 11, 13, 14, and 15 act as “buffer” blocks isolating the test block from the training blocks, thereby ensuring that they are spatially independent. We iterate through this process with all 23 blocks acting as the test block once and then average the resulting 23 estimated values of RMSE to obtain a single estimated RMSE of the fitted model $\widehat{f}$’s predictive error for the entire study region.

While other implementations of cross-validation exist, in particular implementations that incorporate more principled approaches to determining grid sizes [24], we favor the above blocked approach for its ease of implementation and understanding.

We wrote and fit the model in R [19]. We used the `tidyverse` package for data manipulation and graphing [25]. We used the `ggrdiges` package for graphing [26].

Results

Permutation test and cross-validation results

In Fig 2, we plot the result of the permutation test and spatial cross-validation for the three species-groupings models. In all three cases the identity of the competitor matters; the RMSE with actual competitor identity is less than all RSMEs when the competitor

Fig 2. Comparison of the RMSE for all three species-groupings models. We calculated the RMSE with and without spatial cross-validation. The dotted lines show the RMSEs when the species identity of competitor species were not shuffled. The RMSE calculated from 99 shuffled is shown in the distributions.

Fig 3. Posterior distribution of $\beta_{0,j}$ for the family-level model. These distributions give the estimated baseline growth (cm) for each family.

identity was shuffled (Fig 2 compare dotted horizontal lines to histograms). To address the second question, which species-grouping does the best job of describing competition, compare between subpanels in Fig 2. The trait-grouping model performed much worse than the two phylogenetic groups models. Without spatial cross-validation the species level grouping greatly out performs the other two models, but with cross-validation the species and family models perform just as well. We will use the family-level model for the remainder of the paper, as it performs just as well as the species-level one with many fewer parameters.

Posterior distributions of parameters of best model

We find that the family-level model performed the best, so here we analyze the posterior distributions of relevant ($\boldsymbol{\beta}_0, \boldsymbol{\beta}_{\text{dbh}}, \boldsymbol{\lambda}$) parameters for this model. In Fig 3, we plot the posterior distribution of all $\beta_{0,j}$ baselines for all families, j. These values range from 0.05 to 0.4 cm/yr. We generally have a better estimate for the value for families with a larger sample size. In Fig 4, we plot the posterior distribution of all $\beta_{\text{dbh},j}$. This is the effect of tree DBH on growth rate. For most families these values are between 0 and 0.05. For a few families the estimate is negative.

In Fig 5, we plot the posterior distribution of all $\boldsymbol{\lambda}$ relating to inter-family competition. For clarity this figure shows the $\boldsymbol{\lambda}$ values for the eight families with at least 200 individuals in the plot (for the full 20-by-20 $\boldsymbol{\lambda}$ matrix see S1 Fig). Fig 5 shows differences in family-level *effect* and *response* to competition. Some families, such as Fagaceae and Juglandaceae, have a strong negative effect on the growth of all other families. Other families, such as Rosacease, have a positive effect on the growth of most other families. Generally there is a strong negative intra-family affect even for families which have little negative or even a positive effect on other families, for example Ulmaceae and Sapindaceae.

Spatial patterns in residuals

In Fig 6, we plot the spatial pattern of the residuals for the family-level model. There is clear spatial patterning to these residuals, with clusters of trees growing faster than the model predicts and other clusters growing slower. This suggests some spatial factor beyond competition, such as soil fertility, is important to determining tree growth.

Discussion

Here we test whether species identity matters for competitive interactions among forest trees. We find a strong signal that identity matters for both the effect focal trees feel

Fig 4. Posterior distribution of $\beta_{\text{dbh},j}$ for the family-level model. These distributions give the estimated increase in annual growth (cm) per DBH (cm).

Fig 5. Posterior distribution of lambda values, family-specific competition coefficients. Read across rows for that family's competitive *effect* on other families and down columns for how a family *responses* to the competition of other families. Here we just show the 8 families for which there are at least 200 individuals in the plot. For a high resolution version of this image for the full 20-by-20 lambda matrix see see S1 Fig.

Fig 6. Spatial pattern of residuals for the family-level model. Predicted growth calculated by training the model on the data set minus focal tree hectare and buffer. Model 6 considers each individual's family and the family-level competitive interactions. The residuals show clear spatial patterning.

from competition and the effect competitors exerts on a focal tree. Models which included tree identity outperformed those with randomized identity. This importance of competitor identity has been observed before [3, 8, 13]. Surprisingly we found that the model which predicted the effect of competition best was at the family-level rather than species or trait grouping level. Previous work has found that species traits ~~do a good job of~~ predicting competitive ability [12, 13]. We suspect that this family-level grouping for competition is not a general phenomenon, but a consequence of the particular community in this forest. The forest is undergoing rapid successional change as the oak-hickory overstory is being replaced by more mesic, shade-tolerant species [16, 27]. Oaks and hickories here fill a specific functional role, and we suspect the family-level grouping fit best as a result since it groups many species into these two families.

Here we measured the full $\boldsymbol{\lambda}$ matrix of family-level competitive interactions, which because of our Bayesian approach we can measure all values ~~of~~. This matrix shows clear family-level differences in both the *effect* and *response* to competition. Interestingly, some families have a positive effect on the growth of neighbors, such as Ulmaceae. This is probably not a direct positive effect, but a reflection of the spatial pattern of Ulmaceae individuals. These individuals could inhabit more productive soils, so nearby individuals grow faster. This ~~illustrations~~ a limitation of neighborhood-based methods for measuring competition [4].

One clear pattern seen is that oaks and hickories are particularly strong competitors. Their neighbors have consistently lower growth rates. This is interesting in light of the successional change going on in the forest. Allen et al. [27] show that canopy oaks are rapidly being replaced by red maples and black cherries. Even though we find here that oaks have very strong negative effect on neighbor's growth, that is not enough to have them maintain canopy dominance [27].

We fit Bayesian linear regression models of the growth of a focal tree as a function of its species, its size, and the biomass of the competitors. While more complicated models of growth exist than the one proposed in Eq (1), this particular model has the benefit of having closed-form analytic solutions and thus we can avoid computationally expensive methods for posterior estimation such as Markov chain Monte Carlo. This is of particular importance given the large number of times we fit models when performing our permutation test as well as our spatial cross-validation algorithm.

We highlight the importance of cross-validation. It initially appeared in our non cross-validated error estimates in Fig 2 that the species-level model out performed the family-level model. However, using cross-validation to generate our estimates of model error, both models roughly performed the same. This is due to the over-fitting induced by the large number of parameters of the more complex species-level model. Furthermore, had we not incorporated the inherent spatial structure of our data to our cross-validation algorithm, our model error estimates would have been overly-optimistic. This is a point that been demonstrated in other ecological settings ~~before~~ [23, 28, 29].

Here we provide a flexible method to estimate species-specific competitive effects

Fig 7. Posterior distribution of lambda values, family-specific competition coefficients. Read across rows for that family's competitive *effect* on other families and down columns for how a family *responses* to the competition of other families.

between forest trees. This method could be used for other ForestGEO plots with two or more censuses or with USFS FIA data. Comparisons across plots would be particularly powerful to see whether species-specific interactions are general or site-specific. In the future we hope to produce an R package that includes functions to perform the method presented here.

S1 Fig. Posterior distribution of lambda values, family-specific competition coefficients.

Acknowledgments

We thank the individuals who helped census the plot: Omodele Ajagbe, Bob Barretto, Hillary Butterworth, Richard Byler, Vera Chan, Ben Crotte, David Hudson, Lindsay Ford, Katie Gallagher, Jasmine Gramling, Kate Heflick, Rodica Kocur, Carley Kratz, Rachael Lacey, Isaac Levine, Kathleen Parks, Andrew Phillips, Jayna Sames, Margot Sands, John Schroeder, Leah Spaulding, Ethan Strayer, Jordan Trejo, Justin Waraniak, Padhma Venkitapathy, Olivia Velzy, and Ash Zemenick. We also thank Ryan Giordano and Jonathan Che for their help with the statistical methodology. Chris Dick, Ivette Perfecto, and John Vandermeer, with D.A., are PIs of the Big Woods plot. We thank Stuart Davies and Smithsonian staff for guidance in establishing a ForestGEO plot within the E.S. George Reserve.

References

1. Anderson-Teixeira KJ, Davies SJ, Bennett AC, Gonzalez-Akre EB, Muller-Landau HC, Joseph Wright S, et al. CTFS-Forest GEO: a worldwide network monitoring forests in an era of global change. Global change biology. 2015;21(2):528–549.
2. Woudenberg SW, Conkling BL, O'Connell BM, LaPoint EB, Turner JA, Waddell KL. The Forest Inventory and Analysis Database: Database description and users manual version 4.0 for Phase 2. Gen Tech Rep RMRS-GTR-245 Fort Collins, CO: US Department of Agriculture, Forest Service, Rocky Mountain Research Station 336 p. 2010;245.
3. Canham CD, LePage PT, Coates KD. A neighborhood analysis of canopy tree competition: effects of shading versus crowding. Canadian Journal of Forest Research. 2004;34(4):778–787.
4. Canham CD, Uriarte M. Analysis of neighborhood dynamics of forest ecosystems using likelihood methods and modeling. Ecological Applications. 2006;16(1):62–73.
5. Uriarte M, Condit R, Canham CD, Hubbell SP. A spatially explicit model of sapling growth in a tropical forest: does the identity of neighbours matter? Journal of Ecology. 2004;92(2):348–360.
6. Hubbell SP. The Unified Neutral Theory of Biodiversity and Biogeography. Princeton University Press; 2001.

7. Canham CD, Papaik MJ, Uriarte M, McWilliams WH, Jenkins JC, Twery MJ. Neighborhood analyses of canopy tree competition along environmental gradients in New England forests. Ecological applications. 2006;16(2):540–554.

8. Das A. The effect of size and competition on tree growth rate in old-growth coniferous forests. Canadian Journal of Forest Research. 2012;42(11):1983–1995.

9. Tatsumi S, Owari T, Mori AS. Estimating competition coefficients in tree communities: a hierarchical Bayesian approach to neighborhood analysis. Ecosphere. 2016;7(3).

10. Coates KD, Canham CD, LePage PT. Above-versus below-ground competitive effects and responses of a guild of temperate tree species. Journal of Ecology. 2009;97(1):118–130.

11. Tatsumi S, Owari T, Ohkawa A, Nakagawa Y. Bayesian modeling of neighborhood competition in uneven-aged mixed-species stands. Formath. 2013;12:191–209.

12. Uriarte M, Swenson NG, Chazdon RL, Comita LS, John Kress W, Erickson D, et al. Trait similarity, shared ancestry and the structure of neighbourhood interactions in a subtropical wet forest: implications for community assembly. Ecology letters. 2010;13(12):1503–1514.

13. Kunstler G, Falster D, Coomes DA, Hui F, Kooyman RM, Laughlin DC, et al. Plant functional traits have globally consistent effects on competition. Nature. 2016;529(7585):204–207.

14. Stone M. An asymptotic equivalence of choice of model by cross-validation and Akaike's criterion. Journal of the Royal Statistical Society Series B (Methodological). 1977;39(1):44–47.

15. Hastie T, Tibshirani R, Friedman J. The Elements of Statistical Learning. 2nd ed. Springer Series in Statistics. Springer; 2009. Available from: https://web.stanford.edu/~hastie/ElemStatLearn/.

16. Allen D, Dick CW, Burnham R, Perfecto I, Vandermeer J. The Big Woods research plot at the ES George Reserve, Pinckney, MI, USA. In review;.

17. Kattge J, Diaz S, Lavorel S, Prentice IC, Leadley P, Bönisch G, et al. TRY–a global database of plant traits. Global change biology. 2011;17(9):2905–2935.

18. Maechler M, Rousseeuw P, Struyf A, Hubert M, Hornik K. cluster: Cluster Analysis Basics and Extensions; 2017.

19. R Core Team. R: A Language and Environment for Statistical Computing; 2017. Available from: https://www.R-project.org/.

20. Gelman A, Hill J. Data Analysis Using Regression and Multilevel/Hierarchical Models. Analytical Methods for Social Research. Cambridge University Press; 2007. Available from: http://www.stat.columbia.edu/~gelman/arm/.

21. Gelman A, Carlin JB, Stern HS, Dunson DB, Vehtari A, Rubin DB. Bayesian Data Analysis. CRC Texts in Statistical Science. Chapman & Hall; 2013. Available from: http://www.stat.columbia.edu/~gelman/book/.

22. McElreath R. Statistical Rethinking: A Bayesian Course with Examples in R and Stan. CRC Texts in Statistical Science. Chapman & Hall; 2016. Available from: https://xcelab.net/rm/statistical-rethinking/.

23. Roberts DR, Bahn V, Ciuti S, Boyce MS, Elith J, Guillera-Arroita G, et al. Cross-validation strategies for data with temporal, spatial, hierarchical, or phylogenetic structure. Ecography. 2017;40(8):913–929.

24. Valavi R, Elith J, Lahoz-Monfort JJ, Guillera-Arroita G. blockCV: an R package for generating spatially or environmentally separated folds for k-fold cross-validation of species distribution models. bioRxiv. 2018;doi:10.1101/357798.

25. Wickham H. tidyverse: Easily Install and Load the 'Tidyverse'; 2017. Available from: https://CRAN.R-project.org/package=tidyverse.

26. Wilke CO. ggridges: Ridgeline Plots in 'ggplot2'; 2018. Available from: https://CRAN.R-project.org/package=ggridges.

27. Allen D, Dick CW, Strayer E, Perfecto I, Vandermeer J. Scale and strength of oak–mesophyte interactions in a transitional oak–hickory forest. Canadian Journal of Forest Research. 2018;48(11):1366–1372.

28. Le Rest K, Pinaud D, Monestiez P, Chadoeuf J, Bretagnolle V. Spatial leave-one-out cross-validation for variable selection in the presence of spatial autocorrelation. Global ecology and biogeography. 2014;23(7):811–820.

29. Pohjankukka J, Pahikkala T, Nevalainen P, Heikkonen J. Estimating the prediction performance of spatial models via spatial k-fold cross validation. International Journal of Geographical Information Science. 2017;31(10):2001–2019.

Figure 1 Click here to download Figure fig1-1.pdf

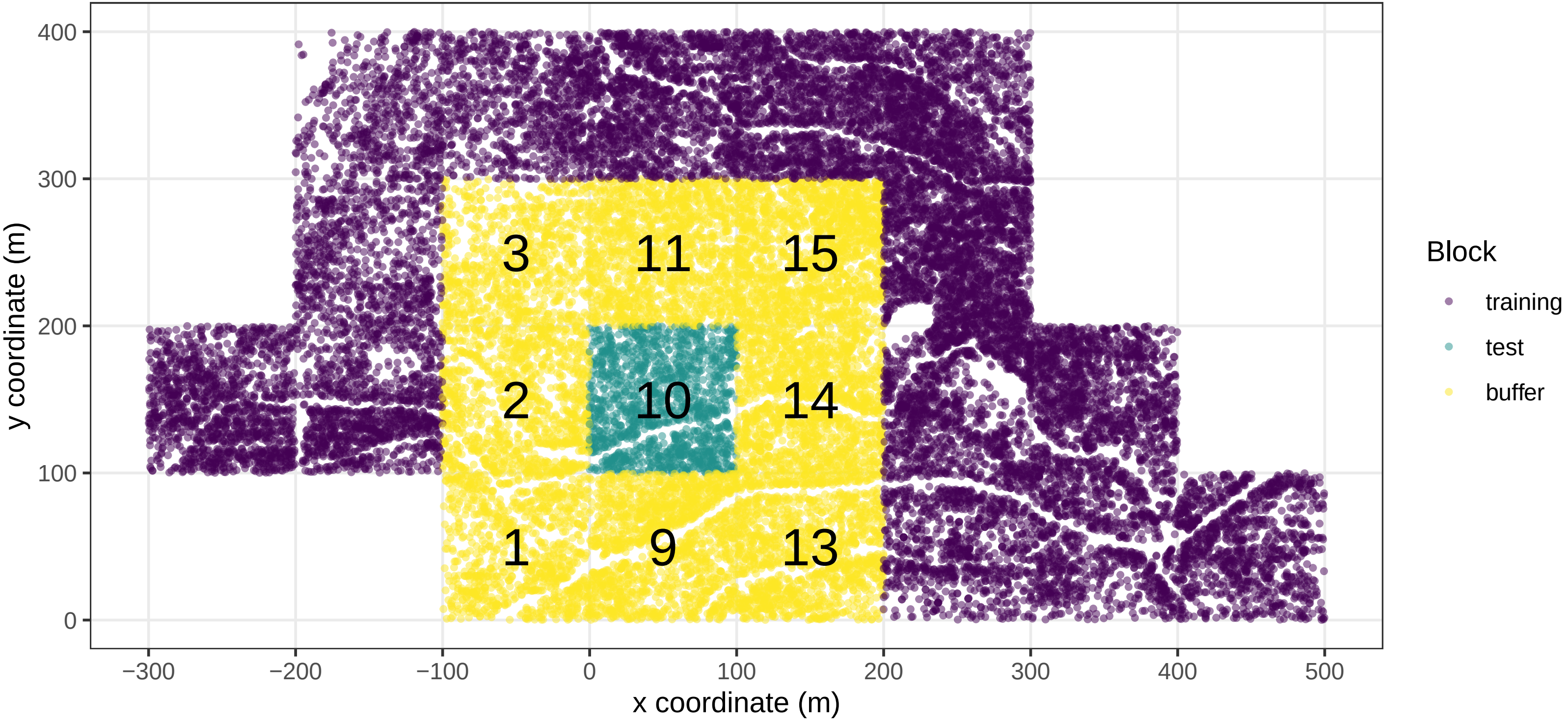

Figure 2

Click here to download Figure fig2-1.pdf

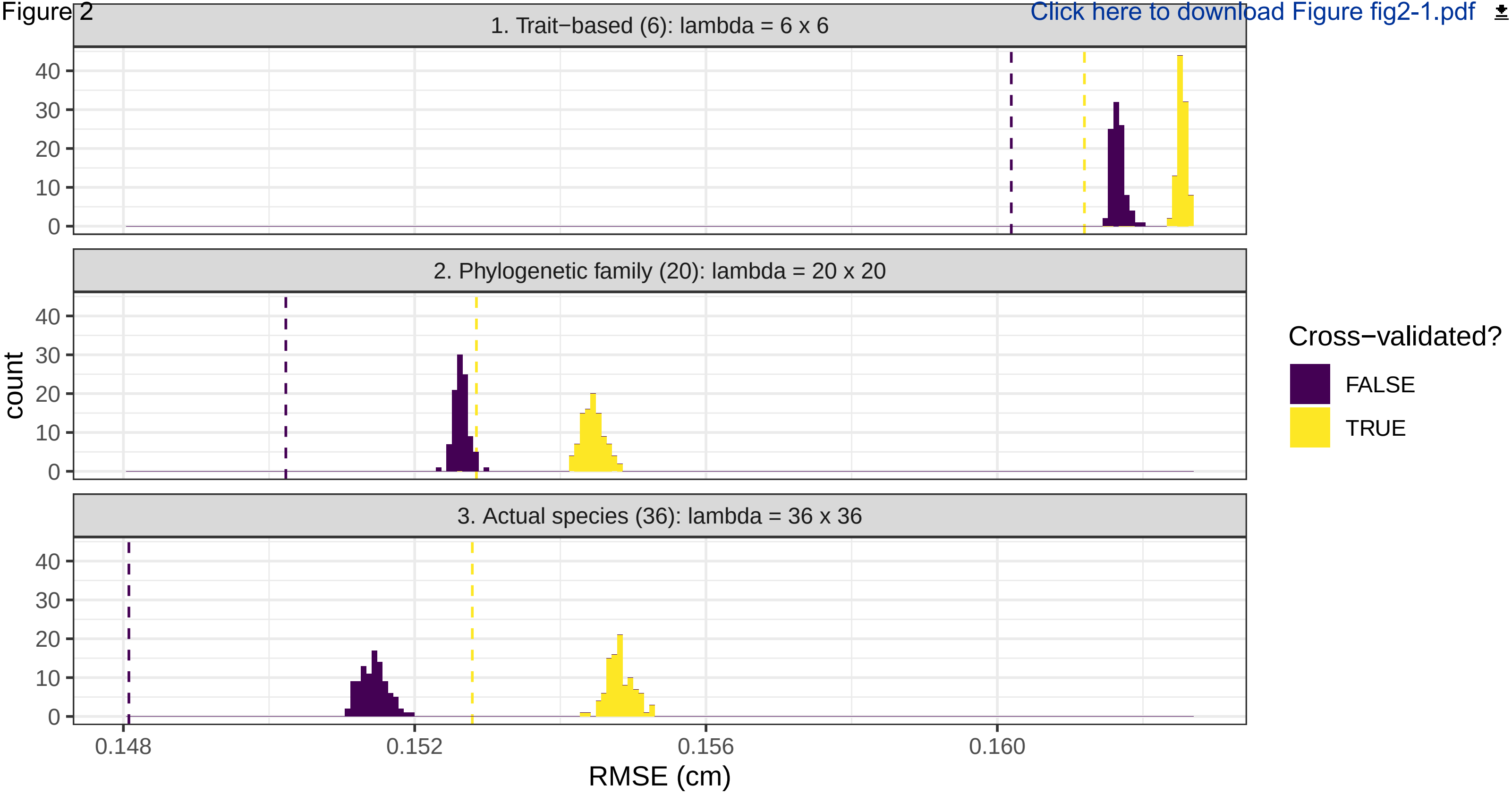

Figure 3

Click here to download Figure fig3-1.pdf

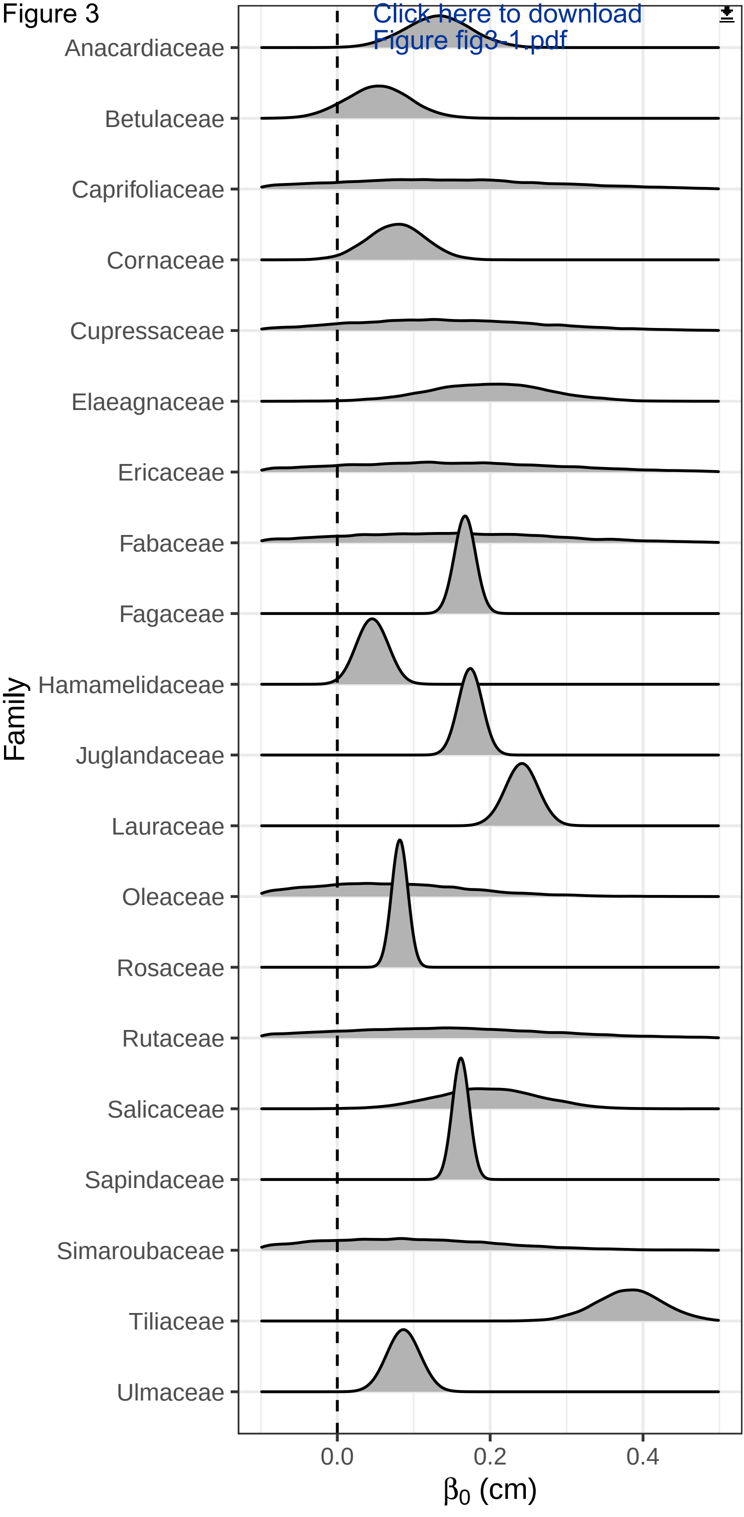

Figure 4

Click here to download Figure fig4-1.pdf

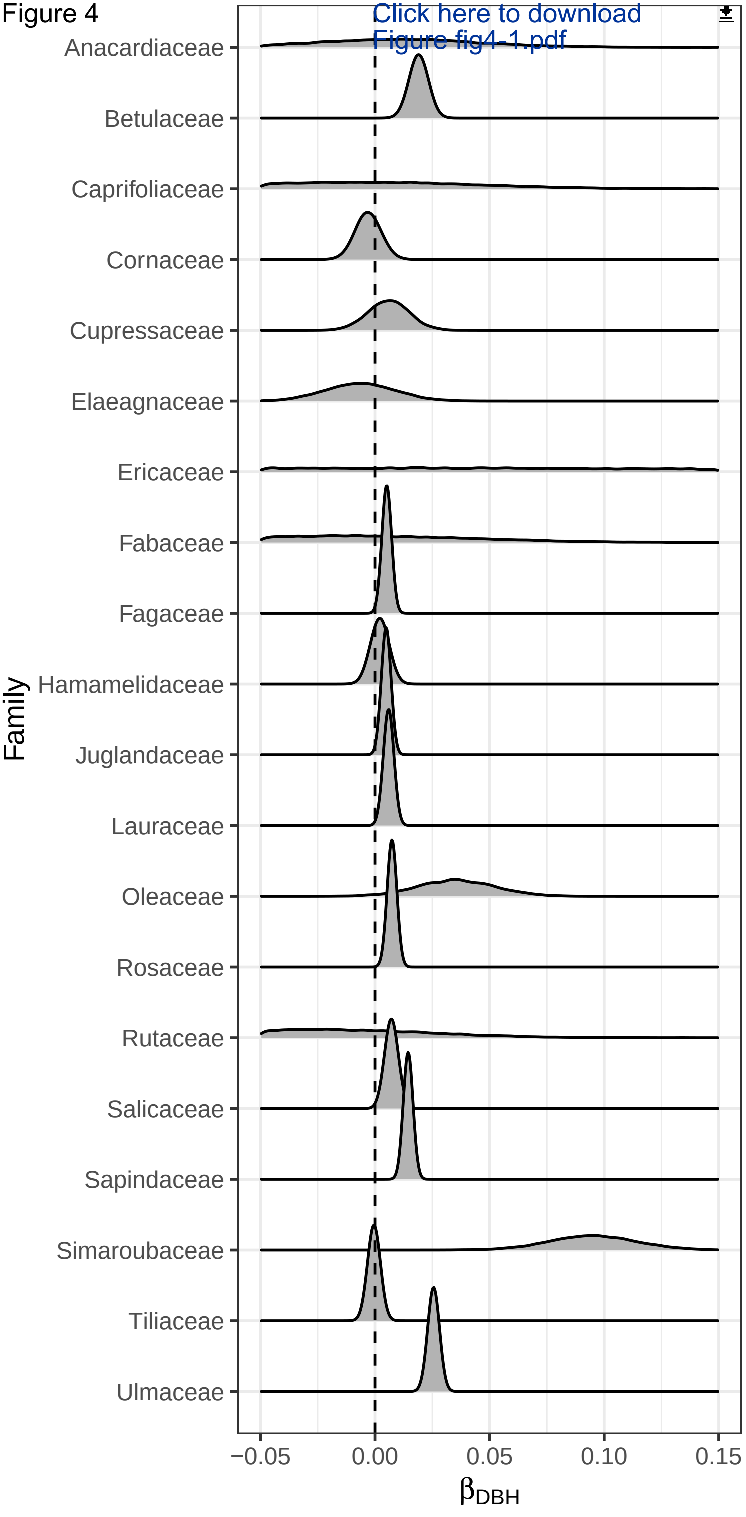

Figure 5

Click here to download Figure fig5-1.pdf

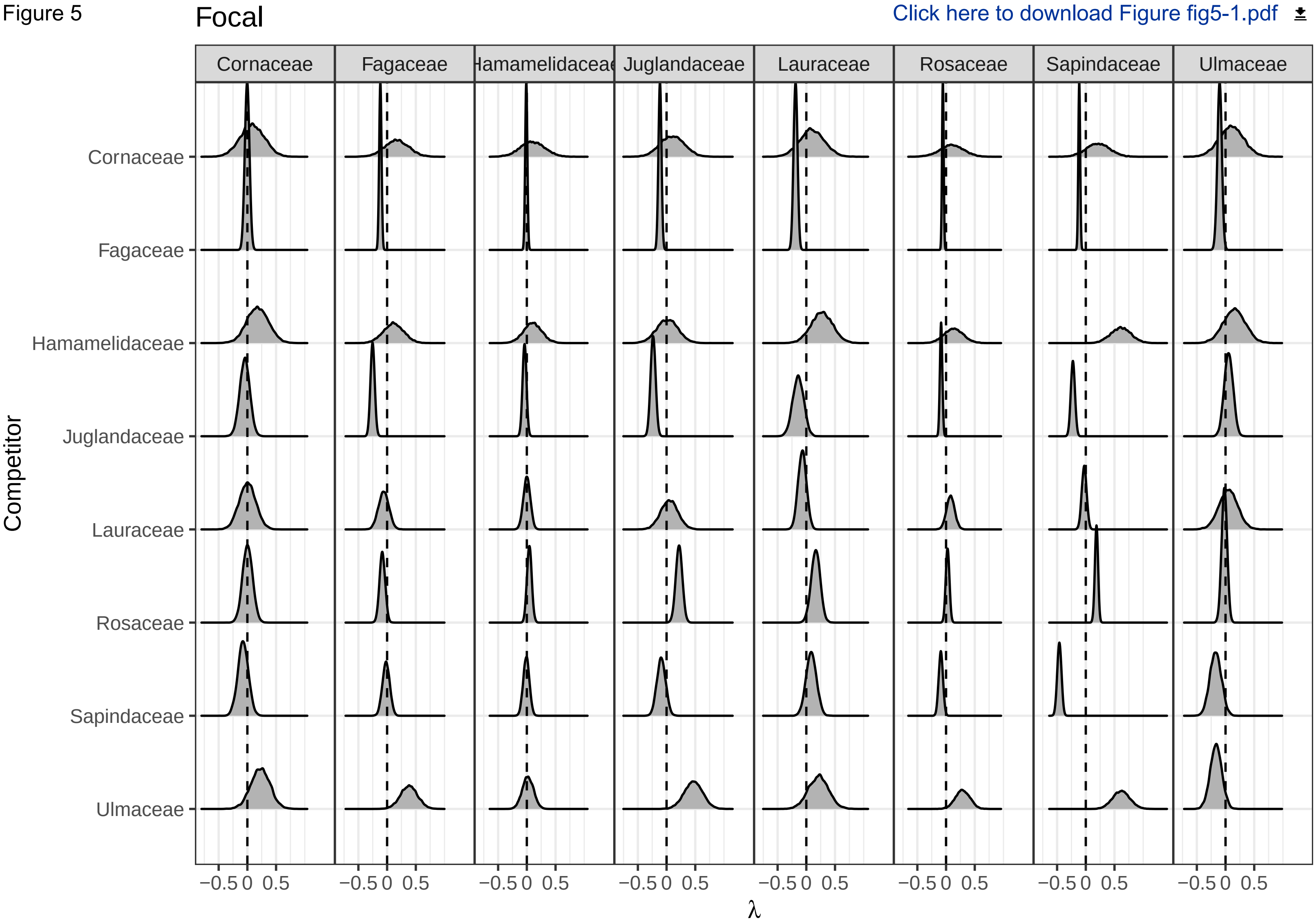

Figure 6

Click here to download Figure fig6-1.pdf

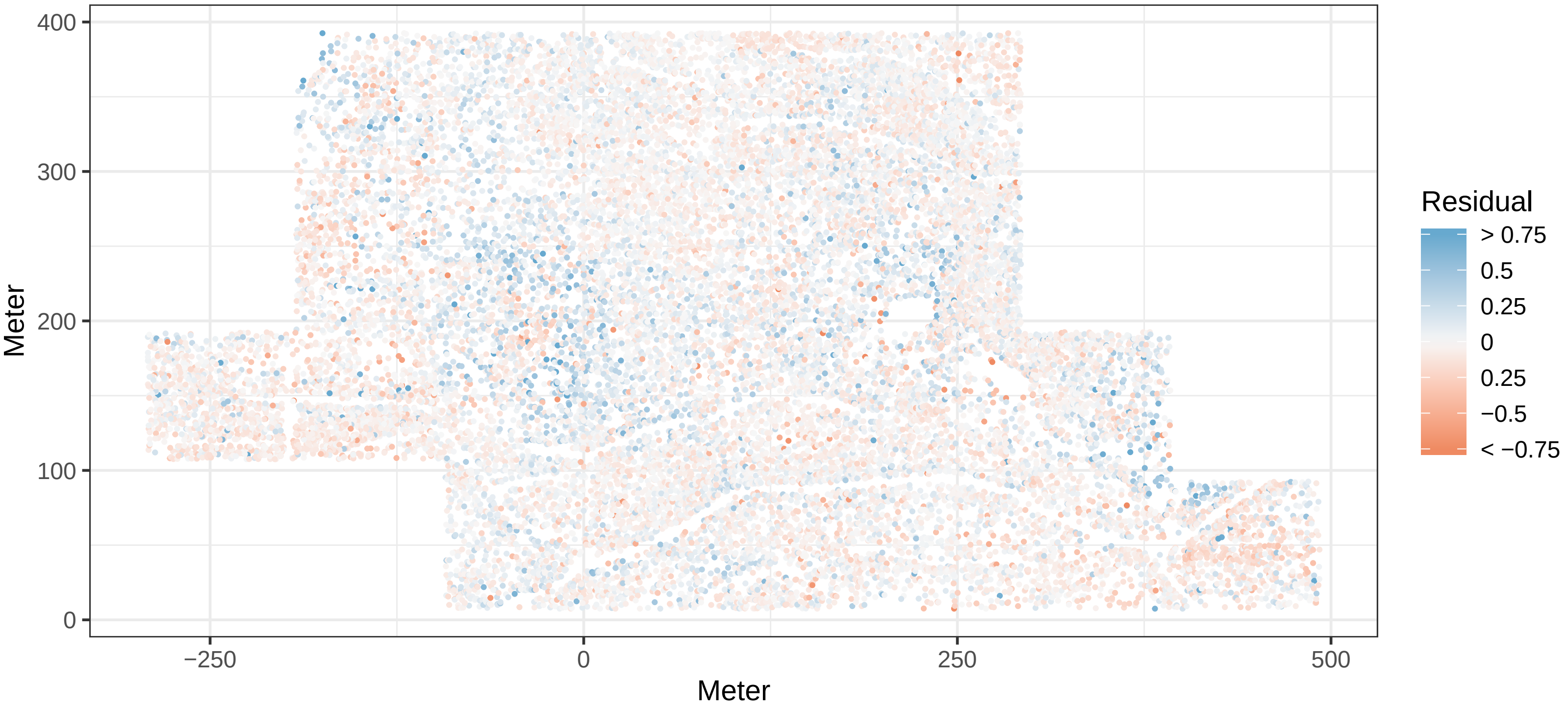

Figure S1

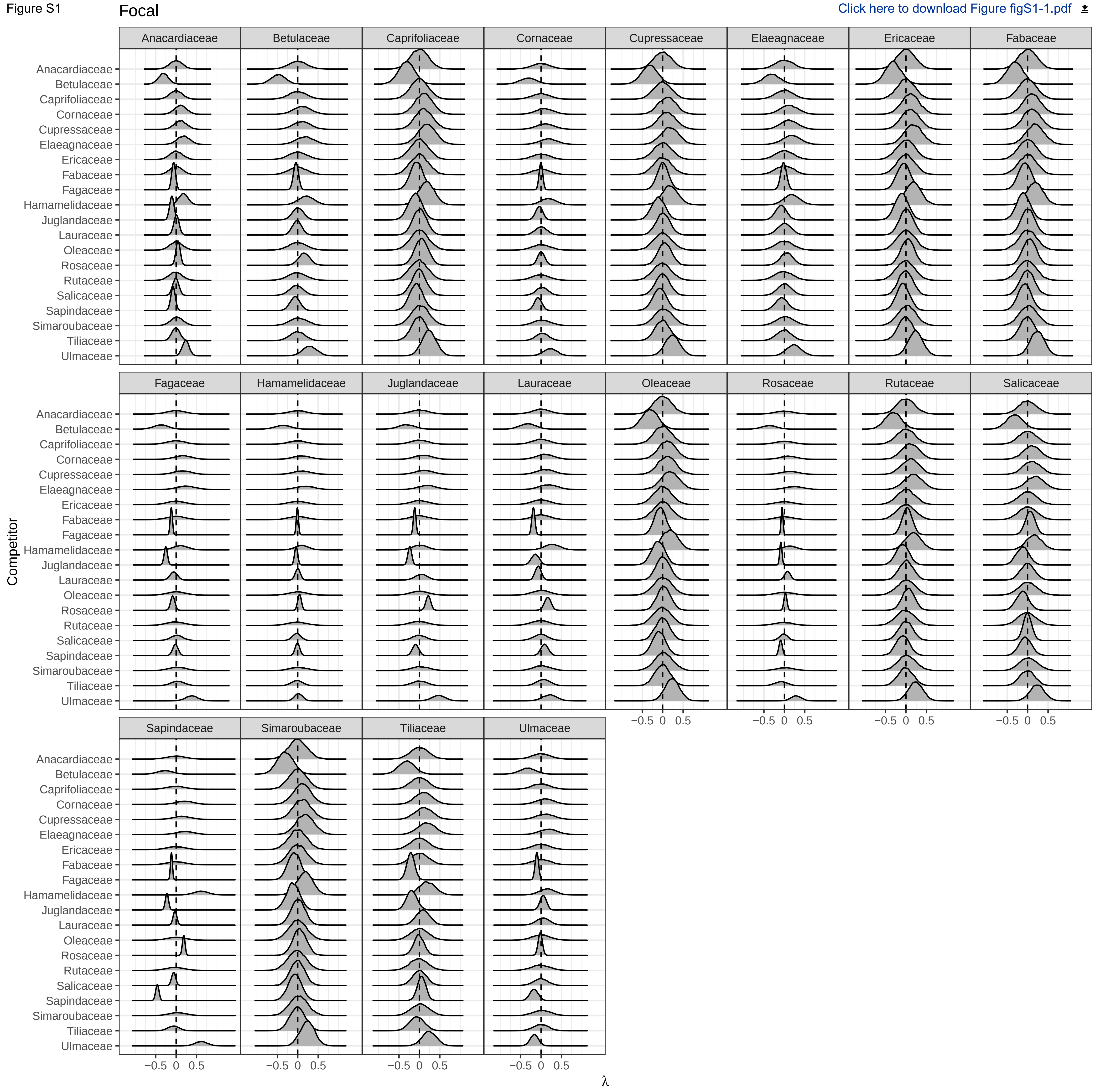

Click here to download Figure figS1-1.pdf

Click here to access/download
Supporting Information
just_SI.pdf
