# Supplementary material for: A permutation test and spatial cross-validation approach to assess models of interspecific competition between trees
Source: PLoS One. 2020 Mar 11;15(3):e0229930. doi: 10.1371/journal.pone.0229930 (PMC7065802; doi:10.1371/journal.pone.0229930)
Supplement: Attachment — Submitted filename: Response to Reviewers.pdf [file pone.0229930.s007.pdf]

December 26, 2019

Dear *PLOS One* Editor:

Thank you for the opportunity to revise and resubmit our manuscript (PONE-D-19-24831). We also thank the reviewers for their thoughtful comments. We have addressed these comments and think that the manuscript is much stronger as a result.

- The data used in this manuscript are now available on the University of Michigan data repository Deep Blue Data. They have a DOI which is linked in the article. https://doi.org/10.7302/wx55-kt18
- We have addressed the grammar and wording suggests of the reviewers. This includes making sure that the manuscript is written in the past tense.
- There was no Figure 7, this was a mistake in the latex code which referred to a figure that didn't exist. We have removed this reference to the nonexistent figure.
- As suggested by reviewer 1, we have added a species list (Appendix 2) and phylogenetic tree of the families in the plot (Figure S2).
- We have explicitly defined the parameters — $\beta_{0,j}, \beta_{\text{DBH},j}, \lambda_{j,k}$ — when we introduce them in the methods section. We made interpretation of them clearer by explaining the fit values in figure legends and the results section. We clearly stated the unit of each parameter (e.g, $\beta_{0,j}$ is in cm y^{-1}). For $\lambda_{j,k}$ we discussed what it means for this parameter to be positive versus negative.
- As suggested by both reviewers, we make more clear what we mean by the spatial pattern of residuals in Figure 6.
- As suggested by reviewer 1, we have added some discussion on how to overcome the limitation of neighborhood-based approaches for measuring competition.
- Reviewer 2 suggested that we remove the last two paragraphs from the introduction. The second to last paragraph summarized our methods and the last paragraph summarized our results. We feel that this summary of methods and results in the introduction helps the reader understand the manuscript as a whole. By giving a summary of the methods and results the reader is better able to understand as they go through the manuscript. We prefer including this end-of-introduction summary, but if the reviewers and editor feels strongly that it should be removed we will.
- Reviewer 2 wanted a link to Allen et al. (2019) (line 80 of the revised manuscript). That article is currently in revisions. We think that it will

be published by the time this current manuscript is published, so can be updated with final citation. If not we will provide some other access to the manuscript draft. We are happy to share a copy with the reviewer before that if requested.

- We have provided justification for our use of 7.5 m as the distance for the competitive neighborhood. See lines 115–116 of the revised manuscript.
- We have included the sample sizes in Figures 3 and 4. Furthermore, we have included an additional figure of the sample sizes of the focal and competitor families pairs (Figure S3); this will facilitate the interpretation of Figure 5.

Thanks again for the opportunity to resubmit this manuscript. If you have any questions, do not hesitate to ask me.

Thanks,

David Allen
Assistant Professor
Department of Biology
Middlebury College
dallen@middlebury.edu
